# GDF9 polymorphisms: influence on ovarian response in women undergoing controlled ovarian hyperstimulation

**DOI:** 10.5935/1518-0557.20200027

**Published:** 2020

**Authors:** João Paolo Bilibio, Arivaldo José Conceição Meireles, Emily De Conto, Panila Longhi Lorenzzoni, Fábio Costa do Nascimento, João Sabino da Cunha-Filho

**Affiliations:** 1 Department of Obstetrics and Gynecology, Universidade Federal do Pará, Belém, PA, Brazil; 2 Clinica de reprodução assistida Pronatus, Belém, PA, Brazil; 3 Programa de Pós-Graduação de Ciências Médicas da Universidade Federal do Rio Grande do Sul, Porto Alegre, RS, Brazil; 4 Clínica Insemine de Medicina Reprodutiva, Porto Alegre, RS, Brazil

**Keywords:** GDF9, polymorphism, *in vitro* fertilization, oocyte, follicle retrieval

## Abstract

**Objective:**

The study looked into the possible influence of GDF9 polymorphisms on ovarian response in women with a normal ovarian reserve undergoing controlled ovarian hyperstimulation for in vitro fertilization (IVF).

**Methods:**

This cross-sectional study included 67 women with normal ovarian reserve aged 30-39 years submitted to controlled ovarian hyperstimulation for IVF. We sequenced four polymorphisms in the GDF9 gene (C398G, C447T, G546A, and G646A) and analyzed their influence on follicular and oocyte outcomes.

**Results:**

The mutant allele C398G decreased the total number of follicles >17mm (6.49 *vs.* 4.33, *p*=0.001), total number of follicles (10.11 *vs.* 7.33, *p*=0.032), number of MII oocytes retrieved, and serum progesterone levels on trigger day. The C447T polymorphism was associated with a greater number of follicles between 12 and 14 mm on the day of r-hCG, while the G546A polymorphism was associated with lower serum progesterone levels on trigger day.

**Conclusions:**

GDF9 gene polymorphisms C398G and C447T adversely affected ovarian response in women undergoing controlled ovarian hyperstimulation. These findings show that in addition to playing a role in the early stages of folliculogenesis, GDF9 polymorphisms have an important impact on the final stage of oocyte development.

## INTRODUCTION

*In vitro* fertilization is an efficient assisted reproduction technology used to treat infertility in women. One of the essential elements in successful IVF is the number of eggs produced following controlled ovarian hyperstimulation (COH) (van Loendersloot *et al*., 2014). Several factors regulate follicular growth and depletion; these include members of the transforming growth factor-β (TGFB) super family (Knight & Glister, 2006; Trombly *et al*., 2009; Pangas, 2012). GDF-9, a member of TGFB super family, is an oocyte-derived factor that is preferentially expressed in the oocytes of humans and mice (Chang *et al*., 2016) known to influence follicle growth and depletion rates (Simoni *et al*., 2008; Knight & Glister, 2006; Juengel & McNatty, 2005; Broekmans *et al*., 2009). GDF-9 genetic variants have been associated with abnormal follicular loss and potential premature ovarian failure (POF) (Broekmans *et al*., 2009).

GDF-9 contributes to ovarian folliculogenesis, a process that controls various granulosa cell processes and the ovulation rate. GDF-9 supports the proliferation of granulosa cells and the growth of cumulus cells, the halt of follicular apoptosis, and the growth of oocytes and embryos (Vitt *et al*., 2000; Yan *et al*., 2001; Orisaka *et al*., 2006; Yeo *et al*., 2008). Additionally, GDF9 stimulates granulosa cell proliferation (Vitt *et al*., 2000) and cumulus expansion (Yan *et al*., 2001), inhibits follicular apoptosis (Orisaka *et al*., 2006), and enhances oocyte and embryo development (Yeo *et al*., 2008; Hussein *et al*., 2006). Taken together, these findings suggest that disrupting the GDF9 gene may prevent folliculogenesis and oogenesis, resulting in ovarian failure. Consistent with these results, GDF9 mutations have been related to abnormal reproductive phenotypes in women, including POF (Hanrahan *et al*., 2004; Dixit *et al*., 2005; Chand *et al*., 2006), polycystic ovary syndrome (Sun *et al*., 2010; Wei *et al*., 2014), and dizygotic twinning (Palmer *et al*., 2006).

Single nucleotide polymorphisms (SNPs) of GDF-9 have been correlated with POF, suggesting that these variants may contribute to aberrant follicular development and oocyte loss (Chand *et al*., 2006; Laissue *et al*., 2006; Kovanci *et al*., 2007). Among the SNPs in the GDF9 gene that have been associated with infertility and POF (Kovanci *et al*., 2007), the most notable are C398-9G (Dixit *et al*., 2005), C447T (Dixit *et al*., 2005, Serdyńska-Szuster *et al*., 2016), G546A (Serdyńska-Szuster *et al*., 2016; Wang *et al*., 2010), and G646A (Dixit *et al*., 2005). Few studies regarding the association between GDF9 polymorphisms and controlled ovarian hyperstimulation (COH) outcomes have been published (Wang *et al*., 2010; 2013). The aim of this study was to investigate the possible influence of GDF9 polymorphisms (C398G, C477T, G546A, and G646A) on ovarian response in women with a normal ovarian reserve undergoing COH for IVF.

## MATERIALS AND METHODS

This cross-sectional study included women aged 30-39 years undergoing COH based on an r-FSH and r-GnRH antagonist protocol sequenced for four polymorphisms of the GDF-9 gene (C398-9G, C447T, G546A and G646A). The study was carried out at the Pronatus Assisted Reproduction Center and at the Federal University of Rio Grande do Sul. The National Committee for Ethics and Research with Human Beings and the Ethics Committee of the Hospital de Clínicas de Porto Alegre approved the study and assigned it certificate no. 25525413.0.0000.5327 (Institutional Review Board equivalent).

A total of 67 women were included in the study. Since the study aimed to evaluate women with a normal ovarian reserve (NOR), the following inclusion criteria were used: age between 30 and 39 years, normal ovarian reserve (antral follicle count >5 (Broer *et al*., 2011), AMH >1.0ng/mL (Tal & Seifer, 2017) and FSH <8IU/L at 3^rd^ of menstrual cycle (27), regular cycles, a diagnosis of infertility (more than one year) by tubal factor (confirmed by hysterosalpingography or videolaparoscopy) or by severe male factor (spermogram of <5 million sperm/ml). The following exclusion criteria were applied: polycystic ovary syndrome, presence of only one ovary or other previous ovarian surgery, previous chemotherapy or endometrioma. All patients were evaluated for ovarian reserve on the third day of the menstrual cycle based on antral follicle count (AFC) and FSH, LH, AMH, and E2 levels.

Ovarian stimulation was initiated on the third day of the menstrual cycle with recombinant FSH (Puregon, Organon) and included r-FSH 200 IU/day for the first three days and then 150 IU/day; after Day 6, a GnRH antagonist, Orgalutran (Organon), was administered daily by S.C. (0.25mg/d) until the administration of recombinant human chorionic gonadotropin (rhCG) (Ovidrel 250 mg/0.5ml, Serono). When the majority of the follicles reached 17 mm, oocyte maturation was stimulated with recombinant hCG and on this day all follicles were measured and serum FSH, LH and P_4_ levels were measured. Ultrasound-guided oocyte retrieval was performed after 35 h and, following denudation, the oocytes were categorized as metaphase II (MII), metaphase I (MI), or prophase I (PI) stage.

Whole blood samples were collected and aliquots of 350 µL from each sample were used for genomic DNA extraction using the Easy DNA kit according to manufacturer’s instructions (Invitrogen, UK). For analysis of the GDF-9 gene, the first portion of exon 2 was amplified by polymerase chain reaction (PCR) using the forward (5′ TTGACTTGACTGCCTGTTGTG 3′) and reverse primers (5′ AGCCTGAGCACTTGTGTCATT 3′) described by Kovanci *et al*. (2007) at an annealing temperature of 63ºC. The DNA concentration used was 200 ng/µL (the product obtained was a 491-bp fragment). Amplification was performed using the Veriti® 96-Well Thermal Cycler (Applied Biosystems, USA) with reagents from Invitrogen (UK) and was confirmed by electrophoresis on 1.5% agarose gel. The 491-bp fragments were purified with PEG8000 and NaCl 2.5 M, and the samples were sequenced according to the Sanger method using an ABI 3500 Genetic Analyzer automated sequencer (Applied Biosystems, USA). The reverse primer was used for sequencing at a concentration of 4 pmol/µL, and the results were compared to the reference sequence from NCBI (NM_005260.3).

Observed numbers for each genotype studied (C398-9G, C447T, G546A, and G646A) were compared with expected values to test whether the sample was in the Hardy-Weinberg equilibrium. Data were tested for normality using the Kolmogorov-Smirnov test. Differences between groups for data with a normal distribution were evaluated with the t-test or one-way analysis of variance (ANOVA) and the Bonferroni post-hoc test when indicated. The Mann-Whitney and Kruskal-Wallis tests were used in the analysis of non-parametric data. Qualitative variables were analyzed with the Chi-square test. Statistical significance was set at <0.05. Statistical tests were performed with the Statistical Package for the Social Sciences 23 (SPSS Inc., Chicago, IL).

## RESULTS

Demographic and clinical characteristics of the women included in the study are shown in Table 1. Their mean age was 35.3 years (SD 3.83) and they had been diagnosed with infertility for 4.2 years (SD 3.2). The Hardy-Weinberg equilibrium test results using the chi-square test for GDF9 polymorphisms were as follows: *p*=0.51 for C398G, *p*=0.33 for C447T, and *p*=0.74 for G546A; the women included in the study were homozygous for wild-type (GG) at position 649.

**Table 1:** Demographic and clinical characteristics of women undergoing IVF

Parameter	Mean	SD
**Age (years) **	35.3	3.83
**Infertility (years) **	4.2	3.22
**Menstrual cycle length (days) **	28.9	3.64
**Ethnic origin, N (%)**		
**Caucasian**	29 (43.9%)	
**Hispanic**	36 (54.5%)	
**African-American**	1 (1.5%)	
**Parity (number of children) **	0.48	0.84
**Body mass index (kg/m^2^) **	24.1	3.28
**Weight (kg) **	63.9	9.88
**Height (m) **	1.62	0.06

The influence of the C398G polymorphism in patients undergoing IVF is shown in Table 2 (two patients were excluded due to polymorphism sequencing failure). The presence of the mutant allele (C398G) was associated with a reduction in the total number of follicles >17mm on trigger day (6.49 *vs*. 4.33, *p*=0.001), a lower number of follicles (10.11 *vs*. 7.33, *p*=0.032), a lower number of MII oocytes retrieved (8.84 *vs*. 5.38, *p*=0.017), and lower serum progesterone levels on trigger day (0.96 *vs*. 0.47, *p*=0.003).

**Table 2:** Influence of the C398G polymorphism of the GDF-9 gene in patients undergoing IVF

Parameter	Genotype*			*p* ^ϯ^	Post Hoc	Allele*		*p* ^§^
	CC (44)	CG (18)	GG (3)		*p* ^ŧ^	C (106)	G (24)	
**Menstrual cycle length (days)**	29.2	28.6	26.3	0.406	-	29.1	28.0	0.888
**Body mass index (kg/m^2^)**	24.5	23.5	23.4	0.593	-	24.3	23.5	0.302
**Antral follicle count (N)**	11.5	9.06	9.00	0.214	-	11.1	9.04	0.084
**Days of induction (N)**	9.4	9.7	8.6	0.589	-	9.53	9.50	0.944
**r-FSH (IU)**	1559	1365	1450	0.142	-	1526	1386	0.079
**Endometrial thickness (mm)**	9.7	9.5	9.8	0.929	-	9.75	9.65	0.826
**Follicles >17 mm on trigger day (N)**	6.95	4.22	4.67	0.021	^ǁ^0.022 ^¶^0.844	6.49	4.33	0.001
**Follicles 14-16 mm on trigger day (N)**	3.11	2.11	3.33	0.254	-	2.94	2.42	0.293
**Follicles 12-14 mm on trigger day (N)**	2.41	1.44	1.33	0.120	-	2.25	1.42	0.017
**Total follicles (N)**	10.77	6.89	8.67	0.049	^ǁ^0.049 ^¶^0.873	10.11	7.33	0.032
**Total oocytes (N)**	11.68	7.33	9.00	0.134	-	10.94	7.75	0.048
**MII oocytes (N)**	9.55	5.39	5.33	0.048	^ǁ^0.049 ^¶^0.796	8.84	5.38	0.017
**MI oocytes (N)**	0.60	0.79	0.76	0.151	-	0.69	0.96	0.400
**VG oocytes (N)**	1.10	1.24	0.88	0.964	-	1.12	1.13	0.995
**FSH, 3^rd^ day of menstrual cycle**	6.83	5.02	5.55	0.165	-	5.47	5.99	0.633
**E2, 3^rd^ day of menstrual cycle**	61.35	49.89	56.63	0.729	-	59.21	51.73	0.506
**LH, 3^rd^ day of menstrual cycle**	4.79	4.04	2.76	0.440	-	4.65	3.67	0.180
**AMH**	1.51	2.15	0.56	0.404	-	1.63	1.75	0.802
**LH, on trigger day**	1.47	2.27	0.90	0.351	-	1.93	1.59	0.495
**Progesterone (ng/ml), on trigger day**	1.04	0.54	0.23	0.347	-	0.96	0.47	0.003
**E2, on trigger day**	1024	1043	672	0.629	-	1108	1194	0.678

* C: cytosine; G: guanine

ϯ Analysis of variance (ANOVA)

ŧ Bonferroni test

§ t-test

ǁ Difference between CC and CG

¶ Difference between CC and GG

The results from the evaluation of the effects of C447T polymorphism in patients undergoing IVF are shown in Table 3 (one patient was excluded due to polymorphism sequencing failure). The presence of the mutant allele C447T significantly increased the number of follicles measuring 12-14 mm on trigger day (1.62 *vs*. 2.46, *p*=0.007). 

**Table 3:** Influence of the C447T polymorphism of the GDF-9 gene in patients undergoing IVF

Parameter	Genotype*			*p* ^ϯ^	Post Hoc	Allele*		*p*^§^
	CC (17)	CT (29)	TT (20)		*p* ^ŧ^	C (63)	T (69)	
**Menstrual cycle length (days)**	27.5	30.0	28.5	0.128	-	28.7	29.2	0.518
**Body mass index (kg/m^2^)**	23.8	23.5	25.2	0.229	-	23.7	24.5	0.164
**Antral follicle count (N)**	10.0	9.7	12.4	0.199	-	9.9	11.2	0.139
**Days of induction (N)**	10.2	9.2	9.3	0.151	-	9.78	9.28	0.103
**r-FSH (IU)**	1455	1466	1581	0.457	-	1460	1532	0.239
**Endometrial thickness (mm)**	9.3	9.9	9.7	0.558	-	9.64	9.82	0.586
**Follicles >17 mm on trigger day (N)**	5.53	5.86	6.75	0.574	-	5.68	6.38	0.280
**Follicles 14-16 mm on trigger day (N)**	3.47	2.45	2.85	0.320	-	3.00	2.68	0.413
**Follicles 12-14 mm on trigger day (N)**	1.29	2.00	2.80	0.037	e0.356 f0.033	1.62	2.46	0.007
**Total follicles (N)**	9.76	8.79	10.40	0.637	-	9.32	9.72	0.696
**Total oocytes (N)**	10.47	9.59	11.00	0.822	-	10.06	10.41	0.803
**MII oocytes (N)**	8.41	7.31	9.05	0.644	-	7.90	8.32	0.714
**MI oocytes (N)**	0.76	0.79	0.60	0.842	-	0.78	0.68	0.632
**VG oocytes (N)**	0.88	1.24	1.10	0.795	-	1.05	1.16	0.708
**FSH, 3^rd^ day of menstrual cycle**	5.55	5.02	6.83	0.460	-	5.28	5.99	0.408
**E2, 3^rd^ day of menstrual cycle**	53.34	48.67	75.88	0.188	-	51.09	63.24	0.172
**LH, 3^rd^ day of menstrual cycle**	4.99	4.28	4.33	0.782	-	4.63	4.31	0.578
**AMH**	1.87	1.75	1.23	0.621	-	1.82	1.45	0.341
**LH, on trigger day**	1.56	1.56	1.75	0.951	-	1.56	1.67	0.578
**Progesterone (ng/ml), on trigger day**	0.52	0.78	1.27	0.245	-	0.64	1.06	0.072
**E2, on trigger day**	1320	1072	1172	0.672	-	1199	1129	0.652

* T: thiamine; G: guanine

ϯ Analysis of variance (ANOVA)

ŧ Bonferroni test

§ t-test

^ǁ^Difference between TT and CT

¶ Difference between TT and CC

The influence of the G546A polymorphism in patients undergoing IVF is shown in Table 4. The presence of the mutant allele G546A decreased serum progesterone levels on trigger day (0.92 *vs*. 0.53, *p*=0.025).

**Table 4:** Influence of the G546A polymorphism of the GDF-9 gene in patients undergoing IVF

Parameter	Genotype*			*p* ^ϯ^	Allele*		*p* ^ŧ^
	GG (47)	GA (17)	AA (3)		G (99)	A (33)	
**Menstrual cycle length (days)**	29.2	27.8	29.5	0.491	28.2	29.3	0.408
**Body mass index (kg/m^2^)**	24.4	23.0	24.9	0.354	24.2	23.4	0.336
**Antral follicle count (N)**	10.6	10.5	10.5	0.996	10.6	10.5	0.927
**Days of induction (N)**	9.3	10.0	10.0	0.298	9.41	10.05	0.133
**r-FSH (IU)**	1495	1541	1200	0.436	1502	1476	0.752
**Endometrial thickness (mm)**	9.7	9.6	9.7	0.985	9.74	9.67	0.874
**Follicles >17 mm on trigger day (N)**	5.70	6.88	7.00	0.499	5.88	6.90	0.244
**Follicles 14-16 mm on trigger day (N)**	2.51	3.65	3.50	0.174	2.68	3.62	0.073
**Follicles 12-14 mm on trigger day (N)**	2.02	2.12	2.50	0.926	2.04	2.19	0.720
**Total follicles (N)**	8.81	11.12	13.00	0.270	9.16	11.48	0.097
**Total oocytes (N)**	9.74	11.35	12.50	0.712	9.99	11.57	0.397
**MII oocytes (N)**	7.43	9.82	10.00	0.395	7.79	9.86	0.179
**MI oocytes (N)**	0.68	0.76	1.50	0.618	0.90	0.69	0.444
**VG oocytes (N)**	1.26	0.76	0.50	0.533	0.71	1.18	0.252
**FSH, 3^rd^ day of menstrual cycle**	5.75	5.60	2.60	0.785	5.73	5.22	0.673
**E2, 3^rd^ day of menstrual cycle**	61.68	46.95	43.60	0.557	59.44	46,21	0.271
**LH, 3^rd^ day of menstrual cycle**	4.16	4.36	6.90	0.233	4.19	5.92	0.029
**AMH**	1.58	1.65	2.45	0.862	1.60	1.81	0.686
**LH, on trigger day**	1.85	1.16	1.00	0.446	1.72	1.14	0.224
**Progesterone (ng/ml), on trigger day**	0.99	0.57	0.30	0.491	0.92	0.53	0.025
**E2, on trigger day**	1106	1184	2800	0.093	1119	1386	0.204

* G: guanine; A: adenine

ϯ Analysis of variance (ANOVA)

ŧ t-test

## DISCUSSION

Previous studies have described the negative impacts of GDF9 polymorphisms on ovarian reserve leading to POF (Dixit *et al*., 2005; Chand *et al*., 2006). Since the present study included only patients with a normal ovarian reserve, it provided insight into the effects of GDF-9 polymorphisms on ovarian response to COH in these patients. In other words, it looked into whether these polymorphisms influence not only the ovarian reserve as previously described, but follicular growth as well. We found that the presence of some of the analyzed polymorphisms influenced follicular development or hormone production in some way. The presence of the C398G polymorphism was associated with a lower number of retrieved MII oocytes (8.8 *versus* 5.3), a lower number of total follicles on trigger day (10.1 *versus* 7.3), and lower levels of serum progesterone on trigger day (0.9 *versus* 0.4). The C447T polymorphism was associated with a greater number of follicles between 12 and 14 mm (1.6 *versus* 2.40), indicating it may impair follicular growth. The G546A polymorphism, such as C398G, has also been associated with lower levels of serum progesterone on trigger day (0.92 *versus* 0.53).

*In vitro* studies using recombinant GDF9 protein have clarified the biological roles and importance of GDF9 activity in follicle growth and development in all stages of folliculogenesis. In the preantral stage, GDF9 has been shown to stimulate the growth of *in vitro* cultured preantral follicles (Hayashi *et al*., 1999) and to promote growth of early-preantral follicles in human ovaries (Hreinsson *et al*., 2002). In the transition to the antral stage, it appears that GDF9 promotes follicular survival by suppressing granulosa cell apoptosis and follicular atresia (Orisaka *et al*., 2006). This may be achieved in part by GDF9 stimulating the expression of follicular FSH receptor (FSHR), since adequate FSHR levels in granulosa cells are essential for FSH-dependent antral-follicle growth. These mechanisms explain why the presence of some GDF-9 polymorphisms may influence the ovarian reserve, since they have been associated with diminished ovarian reserve (DOR), poor response after ovarian stimulation, and poor IVF outcome in studies comparing women with infertility and females with a normal ovarian reserve (Wang *et al*., 2010; 2013; Greene *et al*., 2014).

Published data further reinforce the findings of our study. GDF-9 has a role not only in follicular recruitment, but also in the entire process from follicle recruitment to oocyte maturation. We evaluated women with a normal ovarian reserve and no differences in the number of antral follicles; however, the presence of some GDF-9 polymorphisms resulted in altered follicular growth (C447T and C398G) and culminated in fewer retrieved MII oocytes (C398G) and fewer antral follicles.

Studies have demonstrated that many intra- and extra-ovarian factors, including FSH, LH and E_2_, play important roles in the development of follicles; however, paracrine signals derived from oocytes, including GDF9, seem to be the predominant determinants of the developmental state of follicles (Wei *et al*., 2014; Emori & Sugiura, 2014; Li *et al*., 2014). Prior to the LH surge, cumulus cells require GDF9 to support metabolic cascades, such as glycolysis and sterol biosynthesis (Sugiura *et al*., 2005). GDF9 also regulates diverse processes and gene expression during the preovulatory stage (Elvin *et al*., 2000) and enhances cumulus cell expansion in the presence of FSH (Elvin *et al*., 1999), but not in the absence of FSH (Dragovic *et al*., 2005). Beyond that, GDF-9 induces an EP2 signal transduction pathway, which appears to be required for progesterone synthesis in cumulus granulosa cells (Elvin *et al*., 2000). These mechanisms may explain the reason for the decrease in serum progesterone levels associated with the C447T and G546A polymorphisms. Failure of these mechanisms may decrease serum progesterone production, and cause or reflect failures in maturation and oocyte quality.

Importantly, the present study showed a negative association between GDF9 polymorphisms and follicular development in women with a normal ovarian reserve undergoing COH for IVF in an ethnically heterogeneous population. The findings suggested that GDF9 polymorphisms might be used as biomarkers in patients undergoing ovarian COH, since even in patients with a normal ovarian reserve the presence of these polymorphisms indicates a worse prognosis for COH. Additionally, women under gestational counseling may be advised to anticipate pregnancy if have these polymorphisms, since they may suffer with early ovarian reserve decreases. 

## CONCLUSION

We concluded that GDF9 gene polymorphisms (C398G, C477T, G546A) adversely affect ovarian response in women with a normal ovarian reserve undergoing COH for IVF. The presence of the C398G polymorphism was associated with a lower number of retrieved MII oocytes and a lower number of total follicles on r-hCG day. The C447T polymorphism was associated with a greater number of follicles between 12 and 14mm on the day of r-hCG, while the C398G and G546A polymorphisms were associated with lower levels of serum progesterone on r-hCG day. These data show that this member of the TGFB family functions in the early stages of folliculogenesis, causing DOR, and exerts an important influence on the final stage of oocyte development, even in patients with a normal ovarian reserve.
